# Fluorinated aggregated nanocarbon with high discharge voltage as cathode materials for alkali-metal primary batteries

**DOI:** 10.3389/fchem.2024.1484668

**Published:** 2024-10-02

**Authors:** Huixin Chen, Ke Yan, Yan Zou, Qi Xia, Xiaoyu Kang, Hongjun Yue, Ding Chen

**Affiliations:** ^1^ State Key Laboratory of Advanced Design and Manufacturing for Vehicle Body, College of Mechanical and Vehicle Engineering, Hunan University, Changsha, Hunan, China; ^2^ State Key Laboratory of Structural Chemistry, Fujian Institute of Research on the Structure of Matter, Chinese Academy of Sciences, Fuzhou, Fujian, China; ^3^ Xiamen Key Laboratory of Rare Earth Photoelectric Functional Materials, Xiamen Institute of Rare Earth Materials, Haixi institutes, Chinese Academy of Sciences, Xiamen, Fujian, China; ^4^ College of Chemistry, Fuzhou University, Fuzhou, Fujian, China; ^5^ Changsha Ecological Environmental Monitoring Centre of Hunan Province, Changsha, Hunan, China

**Keywords:** alkali-metal/CF_*x*_ primary batteries, aggregated nanocarbon, semi-ionic C-F bonds, high discharge voltage, rate performance

## Abstract

Due to its exceptionally high theoretical energy density, fluorinated carbon has been recognized as a strong contender for the cathode material in lithium primary batteries particularly valued in aerospace and related industries. However, CF_*x*_ cathode with high F/C ratio, which enables higher energy density, often suffer from inadequate rate capability and are unable to satisfy escalating demand. Furthermore, their intrinsic low discharge voltage imposes constraints on their applicability. In this study, a novel and high F/C ratio fluorinated carbon nanomaterials (FNC) enriched with semi-ionic C–F bonds is synthesized at a lower fluorination temperature, using aggregated nanocarbon as the precursor. The increased presence semi-ionic C–F bonds of the FNC enhances conductivity, thereby ameliorating ohmic polarization effects during initial discharge. In addition, the spherical shape and aggregated configuration of FNC facilitate the diffusion of Li^+^ to abundant active sites through continuous paths. Consequently, the FNC exhibits high discharge voltage of 3.15 V at 0.01C and superior rate capability in lithium primary batteries. At a high rate of 20C, power density of 33,694 W kg^–1^ and energy density of 1,250 Wh kg^–1^ are achieved. Moreover, FNC also demonstrates notable electrochemical performance in sodium/potassium-CF_*x*_ primary batteries. This new-type alkali-metal/CF_*x*_ primary batteries exhibit outstanding rate capability, rendering them with vast potential in high-power applications.

## 1 Introduction

The demand for primary batteries, in terms of power density and energy storage, has steadily increased in recent years owing to scientific and technological advancements, particularly in fields such as aerospace and deep-sea exploration (Zhu C. et al., 2022; Lu et al., 2023). The chemical composition of current and next-generation rechargeable batteries based on transition metal oxides, such as NiCO_2_O_4_-NiO(Ramachandran et al., 2023) and CoMn_2_O_4_ (Ramachandran et al., 2022), may not meet the specific energy requirements of fields such as aerospace. The fluorinated carbon (CF_*x*_) materials possess ultrahigh theoretical energy density, a broad range of operating temperature, and a stable operating voltage plateau, rendering them a potentially useful cathode material for lithium primary batteries with excellent performance (Jones et al., 2022; Guérin et al., 2012; Wang et al., 2024). Current researchers often improve the F/C ratio of CF_*x*_ materials through carbon source design and improved fluorination process to obtain higher energy density (Zhang et al., 2009a; Jayasinghe et al., 2014). However, the high F/C ratio of CF_*x*_ exhibits strong C–F binding and limited electric conductivity introduce several disadvantages, such as low operating voltage plateau, poor rate capability, and low power density (Jiang et al., 2021; Zhang et al., 2009a). The development of CF_*x*_ materials possessing high energy density and exceptional rate discharge performance is crucial (Zou et al., 2024).

To achieve this goal, numerous novel CF_*x*_ materials have been synthesized in the past decade using various carbon precursors (Zhang et al., 2010). For instance, high electrical conductivity partly fluorinated graphite sheets were proposed by Yazami et al., the CF_0.78_ achieved a power density of 8,057 W kg^–1^ at 6C. To achieve a breakthrough in this performance, a further increase in the F/C ratio is required (Yazami et al., 2007), as revealed by ^13^C NMR studies. Neeraj Sharma et al. developed fluorinated nanocarbons, investigating the fluorination process of these materials and the connection between electrochemical performance and the type of C–F bond. The electrochemical lithiation process of fluorinated carbon CF_*x*_ (*x* ≥ 0.5) was clarified through the use of *in situ* high-resolution ^19^F NMR, ^7^Li NMR, and TEM investigations. During the discharge process, LiF, CF_*L*_ (L ≤ 0.06), and carbon were formed (Sharma et al., 2021). It has been found that the semi-ionic C–F bond in CF_*x*_ plays a key role in its electrochemical properties CF_*x*_ materials with semi-ionic C–F bonds and a fraction of sp^2^-hybridised C atoms exhibit high electronic conductivity (Zhong et al., 2018; Sato et al., 2004; Zhu D. et al., 2022).

Over the past few decades, nanocarbon has emerged in diverse dimensions ranging from 0D to 3D, eliciting consistent attention and interest from researchers. Carbon nanotubes (CNTs) and graphene, both possess honeycomb lattices, are representative sp^2^ nanocarbons (Jiang et al., 2017). Due to their peculiar atomic networks and exhibit novel properties, they have been widely studied in terms of structure, properties, synthesis, and applications. Fluorinated nanocarbon materials with reducing particle size and shortening ion diffusion pathways, is expected to enhance the electrochemical performance (Ahmad et al., 2015). Reddy et al. found that ball-milled fluorinated graphite exhibited significantly improved discharge plateau, power density, and energy density, attributing it to the reduction of particle size during milling, which facilitates Li^+^ diffusion. By controlling the fluorination process, fluorinated carbon nanotubes were prepared from multi-walled carbon nanotubes, where the inner non-fluorinated graphite layer maintains electron conductivity while the outer fluorinated layer preserves electroactivity during discharge, thus meeting the requirements for higher energy and higher power electrode materials simultaneously (Li et al., 2019). Fluorinated graphite nanosheets with remarkable electrochemical characteristics were precisely synthesized by Yang et al. (2021) using a novel technique that more semi-ionic C–F bonds are introduced at the edge of the graphite nanosheet, thereby enhancing their rate performance. Hence, nanocarbon materials represent a fine choice as a carbon source due to their splendid electrical conductivity and thermodynamic stability.

In recent years, beyond its application in Li/CF_*x*_ primary batteries as the cathode material, various research groups have explored the utilization of CF_*x*_ in other alkali-metal/CF_*x*_ primary batteries, expanding the research scope of CF_*x*_. Among these, the electrochemical properties of CF_*x*_ in sodium primary batteries (SPBs) and potassium primary batteries (PPBs) are anticipated to be comparable to that of Li/CF_*x*_ primary batteries (LPBs), rendering them promising candidates for high-energy battery applications. Zhong et al. employed a fluorinated nanocarbon material as the cathode in K/CF_*x*_ primary batteries, which exhibit stability in charge-discharge cycles but subpar electrochemical performance (Yue et al., 2021). On the other hand, Li et al. synthesized fluorocarbon nanotubes as the cathode in SPBs, demonstrating comparable capacity to Li/CF_*x*_ primary batteries. However, owing to the low sodium potential of the standard electrode (Na^+^/Na, −2.71V *vs*. SHE) and significant ohmic polarization during the initial phase, SPBs were unable to discharge normally at high rates.

Synthesis of CF_*x*_ materials with higher F/C ratios generally require higher fluorination temperatures, facilitating the formation of CF_2_/CF_3_ groups under these conditions. However, the CF_2_/CF_3_ groups show electrochemical inertness in the context of the Li/Li^+^ reaction within the voltage range of 1–3.3 V, leading in extremely low electrical conductivity, which limits their use in ultra-high power density equipment (Leifer et al., 2009). Here, a fluorinated carbon nanomaterial (referred to as FNC) has been developed using aggregated nanocarbon (ANC) as the carbon source and using an improved low temperature fluorination process (≤400°C). By adjusting the fluorination process to control the degree of fluorination, FNC with high F/C ratios ranging from 0.8 ≤ *x* ≤ 1.05 have been prepared (FNC-*x*). The high F/C ratio in FNC also contains abundant semi-ionic C–F bonds, which was demonstrated to advance electric conductivity and reducing ohmic polarization during initial discharge, and the discharge voltage is further increased. The advantage of high discharge voltage can improve the stacking process of FNC in practical applications and is suitable for more scenarios. FNC-1.0 exhibits maximum energy density of 2,144 Wh kg^–1^, and it keeps up an energy density of 1,250 Wh kg^–1^ at 20C (1C = 860 mA g^−1^). In addition, FNC also shows excellent electrochemical performance when employed as cathode for Na and K alkali-metals batteries.

## 2 Experimental

### 2.1 Material synthesis

Nanocarbon was acquired straight from shanghai naiou nano technology Co., Ltd. This kind of nanocarbon is characterized by high compact density, suitable surface area, and readily bonds with F atoms. The following is the synthetic procedure of FNC: a suitable volume of nanocarbon powder was added to a Ni reactor, and fluorination was performed using a NF_3_ gas at a specific pressure and temperature range, divided into four temperature (300, 330, 360°C and 400°C) and pressure (0.15, 0.20, 0.25,0.30 MPa) intervals for the fluorination reaction. The reaction time was at least 3 h to ensure complete reaction. The F/C ratio of the fluorination product increased as the temperature and pressure during fluorination increased, resulting in FNC-0.80, FNC-0.90, FNC-1.00, and FNC-1.05. Using the weighted method, the F/C ratio was computed using the following Equation 1. In the equation, *W*_*c*_ denotes the mass of the carbon source utilized in the experimental setup, Δ*W* represents the differential variation in the mass of the carbon source, specifically measured before and subsequent to the process of fluoridation.

$$\frac{F}{C}=\frac{∆W/19}{W_{c}/12}$$ (1)

### 2.2 Materials characterization

Field Emission Transmission Electron Microscopy (FETEM, JEOL JEM-F200) and Field Emission Scanning Electron Microscopy (FESEM, Apreo S LoVac, Thermo Corporation) were used to investigate the morphology and microstructure of materials. An X-ray diffractometer (Miniflex 600, Rigaku) equipped with Cu Kα radiation (λ = 1.5406 Å, 15 mA, and 40 kV) was used to analyze the crystal structure of the FNC. Chemical bonding investigations were conducted via Fourier Transform Infrared Spectroscopy (Nicolet iS 50, Thermo Corporation), a Thermo Scientific K-Alpha analyzer with a monochromated Al Kα source was used for X-ray photoelectron spectroscopy (XPS) study, while a LabRAM HR Evolution (Horiba) with laser wavelengths of 325 and 532 nm, and a wavenumber range of 200–3,000 cm^–1^, was used for Raman spectroscopy. Before using an Autosorb-iQ2-MP gas adsorption equipment to record N_2_ adsorption-desorption isotherms, the FNC underwent degassing at a temperature of 150°C, with the process continuing until a pressure of 2 mmHg (267 Pa) was attained. To quantify its specific surface area, the Brunauer-Emmett-Teller (BET) methodology was employed.

### 2.3 Electrochemical test

The electrochemical testing was performed through a constant current discharge process. For the cathode electrode fabrication, a slurry mixture consisting of FNC (80 wt.%), carboxymethyl cellulose sodium (10 wt.%), and acetylene black (10 wt.%) was prepared, utilizing deionized water as the solvent. After that, this slurry was spread over aluminum foil. Subsequently, the electrode was dried in a vacuum oven at 90°C for a duration of 12 h in order to remove residual solvents, followed by cutting into 14 mm disks, achieving an active material loading of 1.25 mg cm^–2^.

An evaluation of the electrochemical performance was conducted by assembling a coin cell. Lithium metal was used to create coin cells (CR2016) as the anode material, and 1.0 M LiFSI in propylene dimethoxyethane (DME) and propylene carbonate (PC) (1:1 vol) acting as the electrolyte, alongside Celgard 2500 as the separator. Coin cells (CR2025) were fabricated utilizing sodium and potassium metal as the anode material, with 1.0 M NaPF_6_ in PC and ethylene carbonate (EC) (1:1 vol), and 1.0 M KPF_6_ in ethylene EC and diethyl carbonate (DEC) (1:1 vol), functioning as the electrolyte. Glass microfiber filters were employed as the separator. Assembly occurred within an argon-filled glovebox (H_2_O < 0.01 ppm, O_2_ < 0.01 ppm). Discharge tests were conducted at 25°C utilizing the MIHW-200-160CH (Shenzhen Neware Electronics Co., China) battery testing system, with a cutoff voltage set at 1.5 V.

The cell was discharged at a rate of 1C for 1 min using the galvanostatic intermittent titration technique (GITT), and then it was allowed to relax for 120 min. In the sections that follow, the parameters pertaining to the CF_*x*_ electrode’s Li^+^ diffusion coefficient are presented.

EIS measurements were conducted utilizing a CHI760E electrochemical workstation (Chenhua Instrument Co., China), with a frequency span from 1 MHz to 0.01 Hz, with an amplitude set at 5 mV.

## 3 Results and discussions

### 3.1 Morphological and structural characterization

**FIGURE 1:**
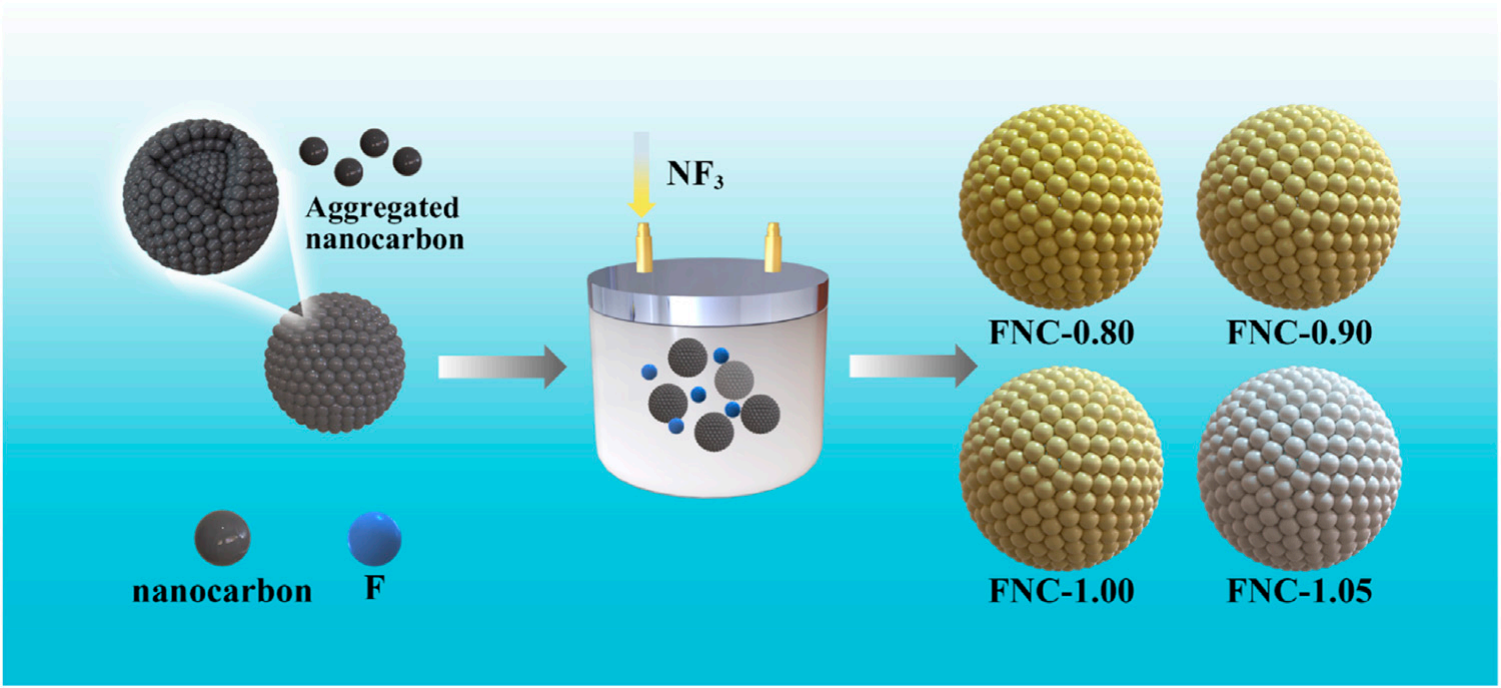
Schematic illustration of the synthesis procedure of the FNC.

Figure 1 depicts the process for preparing FNC, commencing with the introduction of ANC into the reactor, followed by the infusion of NF_3_ for thorough reaction., and the synthesis of FNC with varying F/C ratios was achieved by adjusting the temperature and pressure conditions.

**FIGURE 2:**
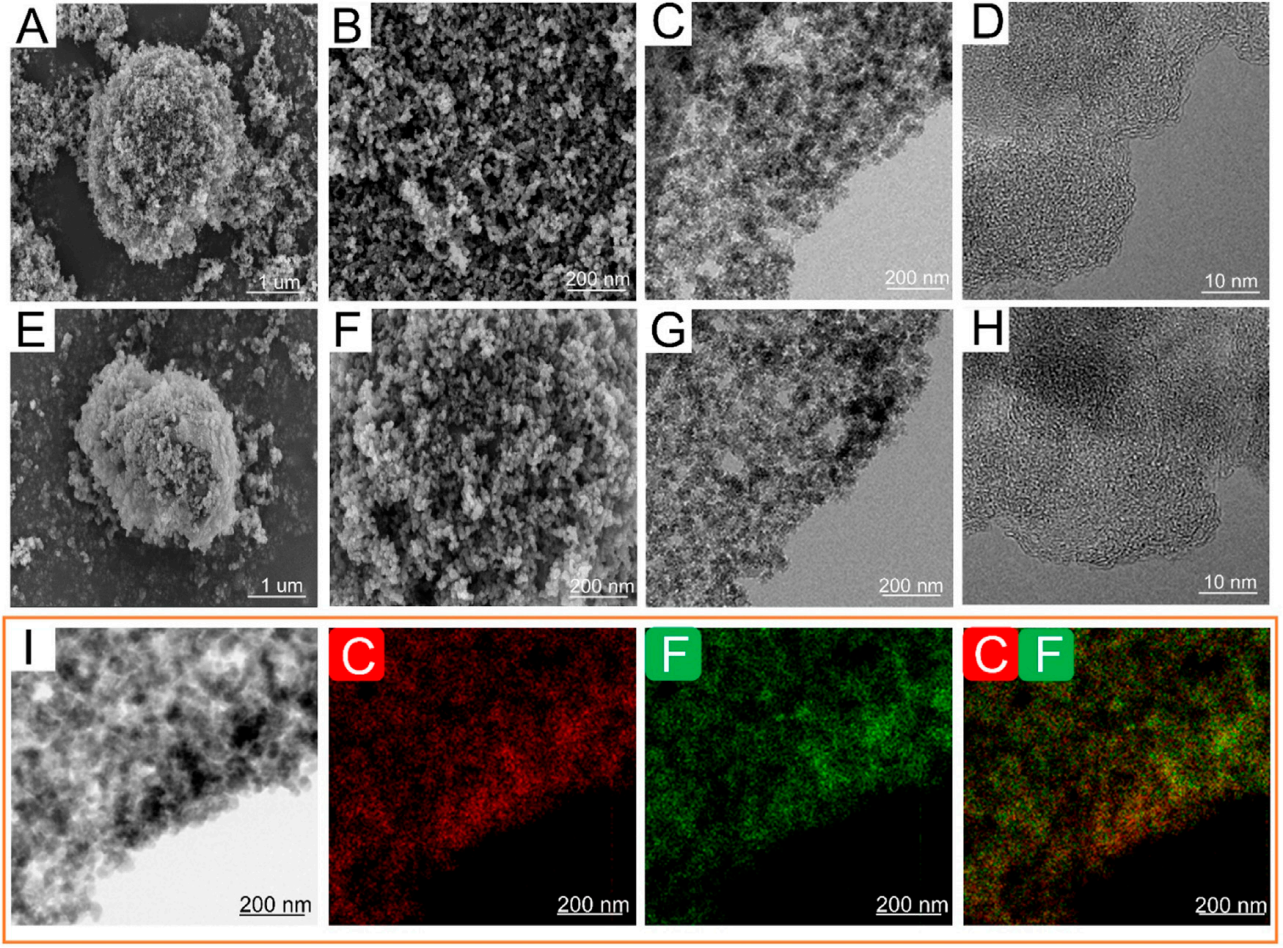
Morphology of ANC and FNC-1.0. SEM images of **(A, B)** ANC, **(E, F)** FNC-1.0. TEM images of **(C, D)** ANC, **(G, H)** FNC-1.0. **(I)** HAADF-STEM image and elemental mapping images of FNC-1.0, elemental distribution of **C** and **F**.

As depicted in Figures 2A, B, numerous spherical nano-carbon particles aggregate to form ANC with sizes ranging from 2 to 5 μm. The aggregate nanocarbon particles exhibit a honeycomb-like structure characterized by a considerable amount of loosely packed voids, which offers unique advantages in terms of physical and chemical properties. Primary ANC particles have a particle size of around 30 nm, according to the TEM picture (Figure 2C), with high disorder observed in the high-resolution view (Figure 2D). Similarly, FNC preserves the original morphology, particle sized and crystalline structure of ANC (Figures 2E–H). The preservation of the inherent structural integrity is crucial, as it facilitates the efficient transport of Li^+^ through the inner of material. Furthermore, the maintenance of the crystalline structure ensures optimal interfacial contact between the F-Li, thereby enhancing the electrochemical performance through improved charge transfer kinetics and effective utilization of active sites. Furthermore, the homogeneous distribution of fluorine and carbon is plainly visible on the FNC-1.0’s high-angle annular dark-field scanning TEM (HAADF-STEM) (Figure 2I). According to the aforementioned findings, the original morphology of ANC was not damaged during fluorination process.

**FIGURE 3:**
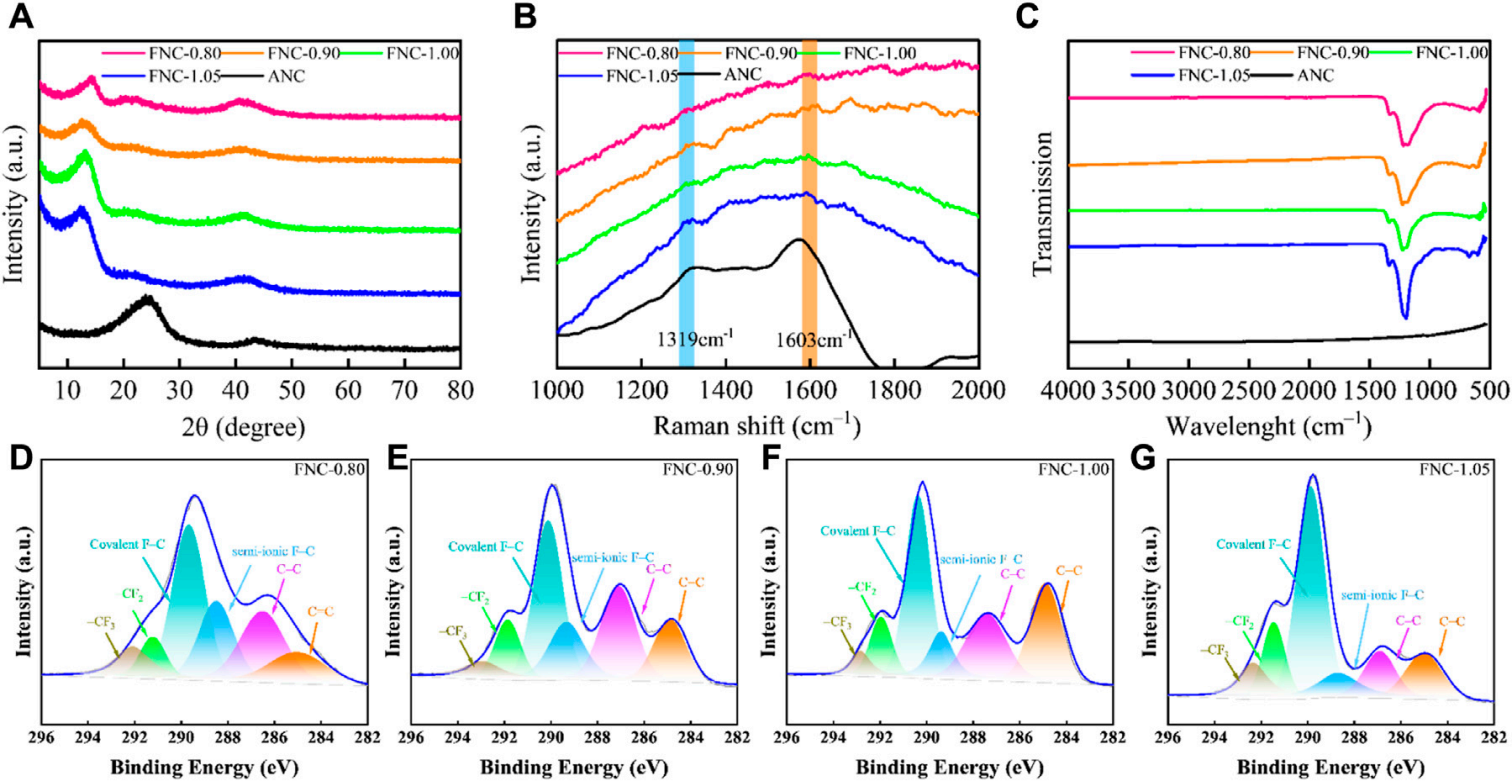
**(A)** XRD patterns of ANC and FNC. **(B)** The Raman spectra of ANC and FNC. **(C)** FTIR spectra of ANC and FNC. **(D–G)** Comparative XPS C 1s spectra of FNC-0.80, FNC-0.90, FNC-1.00, FNC-1.05.

Figure 3A portrays the XRD patterns of raw ANC and FNC materials. The interlayer (002) and (100) reflections cause the ANC material to show notable broad diffraction peaks at 24° and 44°, indicating of a disordered carbon structure. The reflection line in FNC around 13° is the (001) reflection of a high-level fluorinated graphene system, but the markedly reduced intensity and the dramatically broadened width indicate the loss of interlayer correlations between fluorinated carbon sheets (Peng et al., 2019). During fluorination, the peaks associated with the (001) crystal plane exhibit a shift towards a lower angle. This peak shift serves as an indicator of the progressive fluorination depth, manifesting in a gradual enlargement of the interplanar spacing. Concurrently, a reduction in the peak half-width is observed, suggesting a weakening of the interlayer interactions between the FNC layers (Sun et al., 2014). Utilizing the Bragg equation, the calculated interlayer spacings for FNC-0.80, FNC-0.90, FNC-1.00, and FNC-1.05 were determined to be 0.693, 0.703, 0.714, and 0.736 nm, respectively.

The Raman spectrum of ANC (Figure 3B) exhibits two characteristic bands: a defect-induced D band centered at 1,319 cm^–1^ and a crystalline graphite G band centered at 1,603 cm^–1^, indicating the presence of an amorphous structure in ANC (Xu and Buehler, 2009; Rajagopal et al., 2017). The fluorinated FNC possesses pronounced fluorescence capabilities, while the Raman spectrum reveals an absence of distinct Raman bands (Yue et al., 2021). Under the influence of fluorescence, Figure 3B reveals two relatively weak characteristic peaks. Firstly, the peak at 1,323 cm^–1^ corresponds to the D band of carbon materials, the peak at 1,591 cm^–1^ corresponds to the G band, with its shift attributed to the shortened C–C bond lengths.

The chemical composition of ANC and FNC was analyzed using FTIR spectroscopy, as depicted in Figure 3C, to elucidate their respective characteristics. The FTIR spectrum of ANC indicates a lack of active functional groups, with the spectrum showing minimal distinctive features. The utilization of FTIR characterization techniques enabled the investigation of the chemical bonds present in FNC. FTIR characterization was utilized to investigate the chemical bonds of FNC. The detection peaks at 1,341 and 1,222 cm^–1^ signifies the stretching vibration of–CF_2_/CF_3_ groups and covalent C–F bonds, respectively. Furthermore, FNC-0.80, FNC-0.90, and FNC-1.00 consistently exhibit a characteristic peak at 1,160 cm^–1^, which corresponds to the stretching vibration of the semi-ionic C–F bond. This observation provides substantial evidence for the notable concentration of C–F semi-ionic bonds within these materials (Sun et al., 2014; Lee et al., 2003; Guérin et al., 2004).

For the purpose of evaluating the pore structure and quantifying the specific surface area (SSA) of the samples, nitrogen adsorption-desorption isotherms were analyzed at a temperature of 77 K. The nitrogen adsorption-desorption isotherm analysis of ANC and FNC, as depicted in Supplementary Figure S1A, reveals a type II profile without a discernible hysteresis loop throughout the curve, suggesting a non-microporous nature of the materials. The micropore volume of ANC and FNC is very small (Supplementary Table S1), drawing from density functional theory (DFT) calculations, the cumulative pore volumes of micropores for FNC-0.80, FNC-0.90, FNC-1.0, and FNC-1.05 are 0.042, 0.044, 0.035, and 0.032 m^3^ g^–1^, respectively. This further confirms that FNC are non-microporous nanoscale carbon. With the continuous increase in fluorination temperature, the SSA of the corresponding synthesized FNC gradually decreases, which is attributed to the destruction of the FNC structure during the fluorination process. Furthermore, the pore size distribution graph (Supplementary Figure S1B) of ANC and FNC reveals the absence of pores, affirming the non-porous nature of the material.

Additionally, the surface composition of FNC was ascertained by assessing the cumulative intensity of characteristic lines within the XPS spectrum (Supplementary Figure S2). The high-intensity F signal confirmed the successful fluorination of ANC. In alignment with the FTIR analysis findings, the high-resolution C 1s spectra of FNC (Figures 3D–G) validated the existence of carbon-based components with varying compositions and configurations. The deconvoluting of the high-resolution C 1s spectra for the four samples disclosed distinct peaks, with assignments at 284.78, 287.12, 289.11, 290.4, 291.74, and 292.7 eV corresponding to sp^2^ C=C, sp^3^ C–C, semi-ionic C–F bonds, covalent C–F bonds, –CF_2_, and–CF_3_ bonds, respectively (Sun et al., 2014; Robinson et al., 2010; Nair et al., 2010). Upon a comparative analysis of the data, it becomes evident that a substantial fraction of carbon atoms is covalently bonded to fluorine atoms through sp^3^ hybridization. The comprehensive results pertaining to each carbon component are tabulated in Supplementary Table S2. Moreover, quantitative analysis of the high-resolution spectra of F 1s for FNC was performed, and the results are presented in Supplementary Figures S3A–D. Correspondingly, the fittings at 687.35 eV, 688.70 eV, and 690.03 eV correspond to the covalent bonding of fluorine atoms with sp^2^-hybridized carbon atoms, sp^3^-hybridized carbon atoms, and perfluorinated functional groups formed with carbon, respectively. The detailed content and results of various fluorine compositions are provided in Supplementary Table S3. Additionally, the atomic ratio of F/C obtained through XPS analysis was found to be close to the F/C ratio obtained through gravimetric analysis. It is observed that with increasing F/C ratio, the presence of conjugated components in the FNC diminishes, the relative abundance of perfluorinated functional groups steadily increases.

### 3.2 Electrochemical performance

**FIGURE 4:**
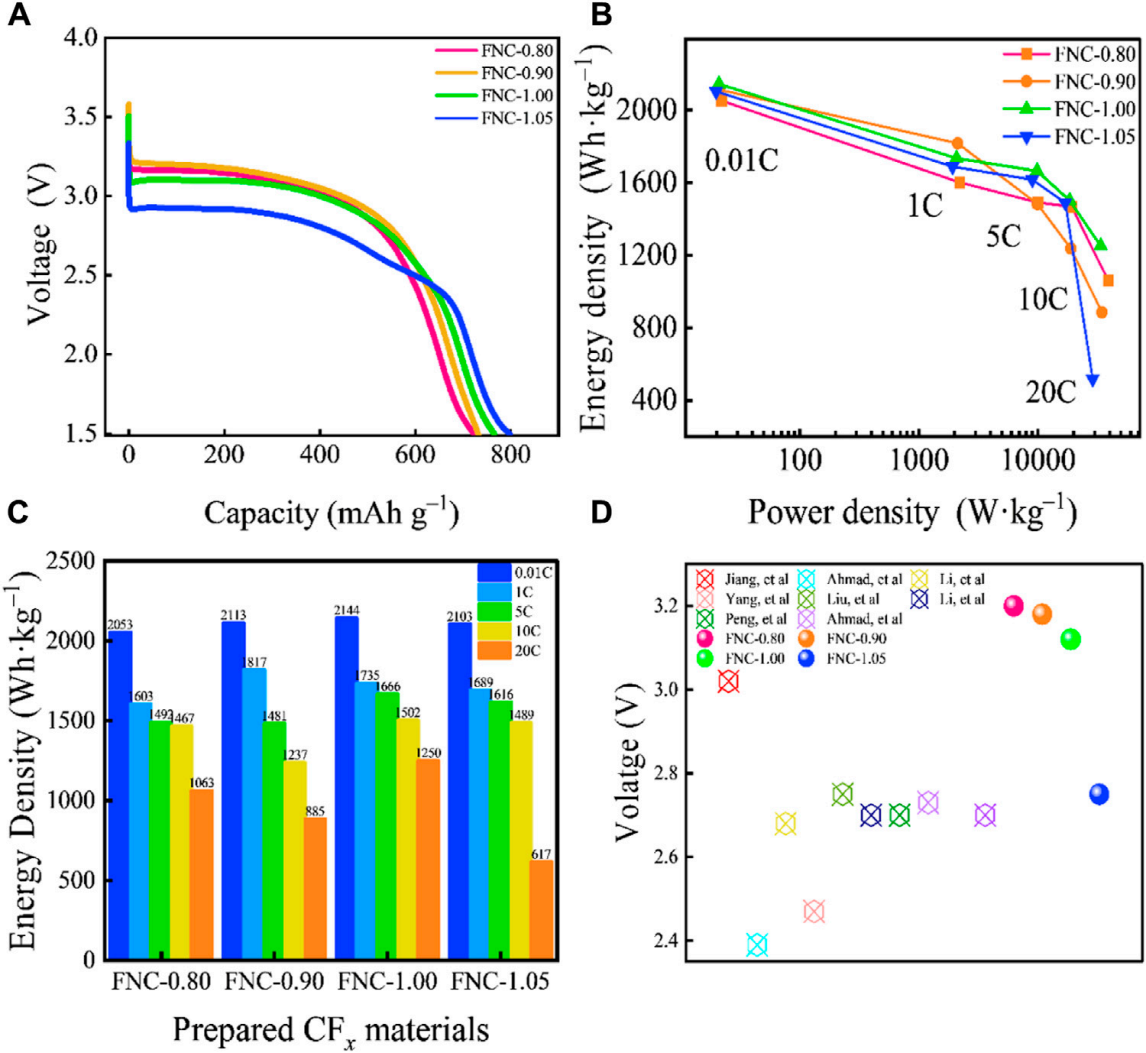
**(A)** Galvanostatic discharge curves at 0.01C rate of FNC. **(B)** Ragone plot of prepared FNC cathodes. **(C)** Diagram of electrochemical performances of prepared FNC cathodes at different discharge rates. **(D)** Comparison of *E*_1/2_ of FNC with previously reported nano CF_*x*_ cathodes. Note: Multiple batteries were simultaneously tested, and all results showed good repeatability.

FNC was assembled as the cathode of a lithium metal primary battery in the form of a coin cell to test and characterize its electrochemical performance. The galvanostatic discharge profiles of FNC at 0.01C are shown in Figure 4A. The specific capacities of FNC-0.80, FNC-0.90, FNC-1.00, and FNC-1.05 are 722, 731, 765, and 798 mA h g^–1^, respectively. Based on the aforementioned data that nearly all C–F bonds participate actively in the discharge process. The corresponding Ragone plot and different rate energy density graph of the prepared FNC cathodes (Figures 4B, C) both demonstrate that FNC has excellent rate capability. Distinct from other nano scaled CF_*x*_ materials, the FNC cathode exhibits superior rate capability and supports high-power output, which is attributed to its aggregated spherical structure, wherein loosely packed and dense voids provide more continuous diffusion pathways for Li^+^ transportation. At 20C, the FNC-1.00 exhibits a power density of 33,694 W kg^–1^ and an energy density of 1,250 Wh kg^–1^. The specific capacity is 578 mA h g^–1^ at 20C, corresponding to a 75.6% retention of the specific capacity at 0.01C.

The F/C ratio exhibited a negative correlation with the discharge voltage but a positive correlation with the specific capacity. Given the higher bond energies of–CF_2_ and–CF_3_ groups, which manifest their inert nature during the discharge process, perfluorinated groups do not contribute to the enhancement of the material’s capacity, but rather decrease the activity of FNC. An increase in the content of perfluorinated groups can exacerbate ohmic polarization and reduce the discharge platform. In addition, high F/C ratio diminishes electrical conductivity, heightens ohmic polarization and lowers discharge voltage (Zhang et al., 2010; Dubois et al., 2012). Consequently, all reported CF_*x*_ products demonstrate distinct voltage delays following discharge. It is observed that the ohmic polarization effect of FNC is relatively weak in the initial discharge stage, and there is no significant voltage delays, contrasting with other CF_*x*_ materials (Supplementary Figure S4) (Watanabe, 1980). In the case of FNC-1.05 with a higher F/C ratio, there is almost no voltage delay phenomenon observed at low discharge rates (<0.5C). As the discharge rate increases (>1C), minor voltage delays emerge in FNC with varying F/C ratios. Even the discharge rate is escalated to 20C, FNC does not exhibit a more severe voltage delay. In addition, the high F/C ratio of FNC presents a higher discharge plateau. The median voltage of the FNC was compared against the discharge median voltages (*E*_1/2_) for all reported nanoscale CF_*x*_ materials (Figure 4D, and detailed information presented in Supplementary Table S4), where in FNC demonstrates a higher discharge voltage (Jiang et al., 2021; Ahmad et al., 2015; Li et al., 2019; Yang et al., 2021; Liu et al., 2023; Li et al., 2021; Peng et al., 2023; Ahmad et al., 2017; Hou et al., 2022). The *E*_1/2_ of FNC are at 3.20, 3.21, 3.13, and 2.88 V (*vs* Li^+^/Li) at 0.01C, which are significantly higher than that of commercial FG (2.71 V *vs* Li^+^/Li) (Chen N. G et al., 2023). FNC-1.0, characterized by a high F/C ratio, and demonstrates a high *E*_1/2_ of 3.13 V. This phenomenon is attributed to the higher content of semi-ionic C–F bonds in FNC, thereby enhancing the electrochemical activity and conductivity of the material.

**FIGURE 5:**
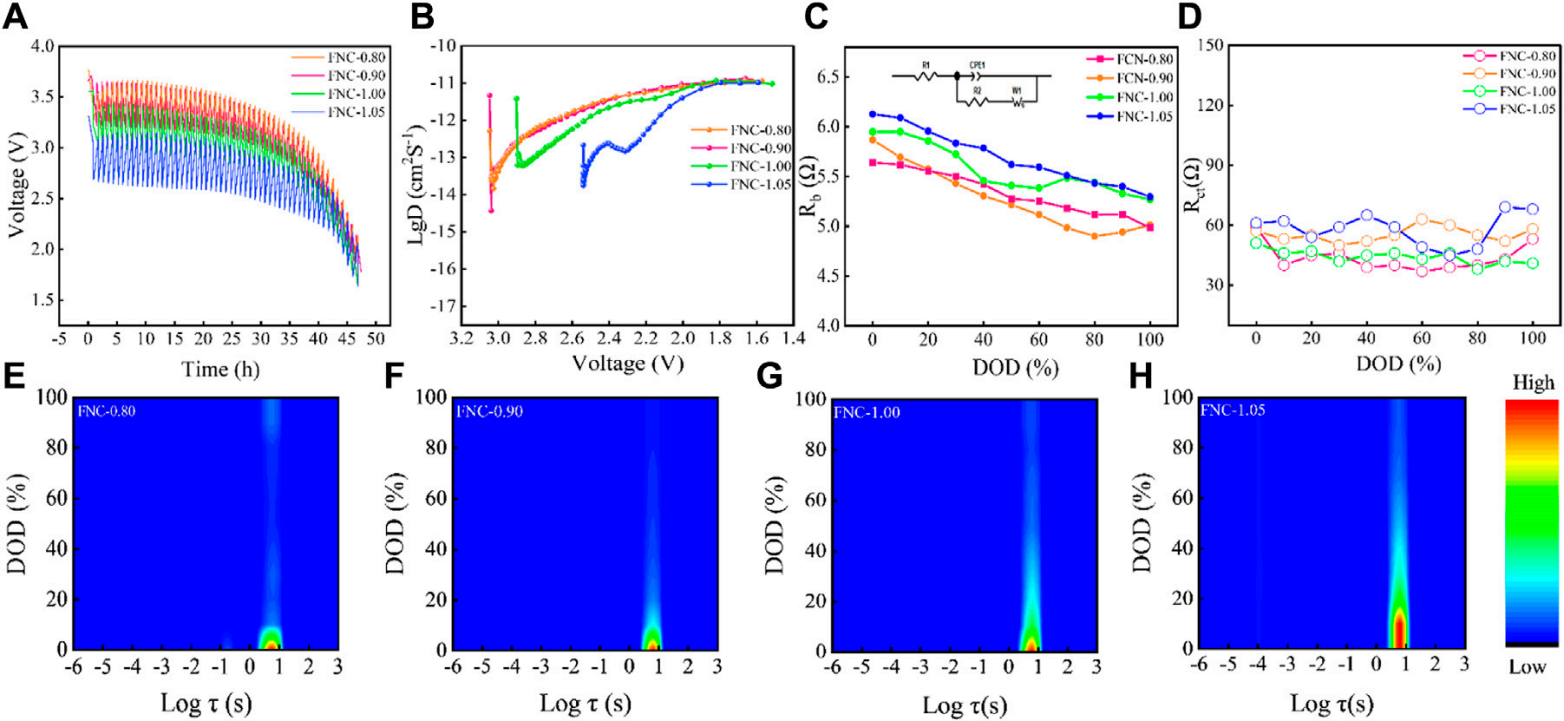
**(A)** Galvanostatic intermittent titration curves of FNC (GITT), **(B)** Li^+^ diffusion coefficient estimated by GITT. **(C)** *R*_b_ variations of FNC under different DOD. **(D)** *R*_ct_ variations of FNC under different DOD. **(E–H)** Impedance intensity map based on DRT under different DOD.

To further validate the rate capability of FNC, the diffusion efficiency of Li^+^ was analyzed using GITT, and the Li^+^ diffusion coefficient (*D*_Li_^+^) was utilized as an assessment metric (Figure 5A). If the applied current is extremely low and the relaxation time is exceedingly brief, the following equation can be used to calculate the *D*_Li_^+^(Deiss, 2005):

DLi+=4ΠI0VmSFZi2dEdδdEdτ2 τ≪L2DLi+ (2)

The chemical diffusion coefficient of the mobile species, denoted as *D*_Li_^+^ (cm^2^ s^–1^), is determined by considering multiple parameters. These include the applied current *I*_0_(A), the molar volume *V*_m_ (cm^3^ mol^–1^), and the thickness *L* (cm) of the electrode material. Additionally, the Faraday constant *F*, the total contact area *S* (cm^2^) between the electrolyte and electrode, and the number of charge transfer *Z*_i_ in the discharge reaction of FNC are also taken into account for the precise calculation of *D*_Li_^+^. Utilizing the Equation 2, the *D*_Li_^+^ was calculated, and Figure 5B demonstrates the gradual variation of *D*_Li_^+^ values during the discharge process. In the initial stage of discharge, the *D*_Li_^+^ value gradually increases due to the generation of carbon from the discharge of CF_*x*_. As the voltage ranges from 3.0 V to 1.5 V, the particle surface progressively becomes saturated with LiF, impeding ion transport, as reflected in the gentle increase trend of *D*_Li_^+^ values. Overall, compared to traditional CF_*x*_ materials, FNC possesses a plethora of highly active semi-ionic C–F bonds, which demonstrate higher *D*_Li_^+^ throughout the entire discharge process, effectively mitigating the voltage delay caused by ohmic polarization. This characteristic contributes to its excellent rate capability. It is noteworthy that the *D*_Li_^+^ value of FNC-1.00 in the 2.5 V–1.5 V range is higher than that of FNC-1.05. These results demonstrate that the average *D*_Li_^+^ value of FNC-1.00 at 1C is higher than that of FNC-1.05, confirms its superior power performance.

The underline mechanism of the excellent electrochemical performance of FNC materials was further analyzed and discussed. Electrochemical impedance spectroscopy (EIS) was recorded for FNC electrode (Supplementary Figure S5A–D) at different depths of discharge (DOD) at 0.1C (cut-off 1.5 V). The Nyquist plot of the FNC cathode exhibits an approximate semicircular feature in the high-frequency region and a slanted line feature in the low-frequency region. This semicircle represents the charge transfer process, and as the discharge depth increases, the decrease in the radius of the semicircle indicates a reduction in the charge transfer resistance (Zhang et al., 2009b; Jiang et al., 2017). Based on the analysis of the equivalent circuit model depicted in the inset of Figure 5C, *R*_b_ signifies the ohmic resistance, while *R*_ct_ denotes the charge transfer resistance, *Z*_w_ represents the Warburg impedance, and CPE stands for the double-layer capacitance (Zhang et al., 2009a; Qian et al., 2022). The *R*_b_ and *R*_ct_ significantly impact the discharge performance of the battery. For materials with a high F/C ratio, the high content of–CF_2_/–CF_3_ groups within the material, which are considered inert fluorine-containing groups, significantly impedes charge transfer (Zhong et al., 2018). These groups, due to their nonconductive nature, impede the transfer of electrons, thereby manifesting a greater charge transfer resistance. Consequently, CF_*x*_ materials with a high F/C ratio exhibit a higher interfacial transfer impedance. With the increase in discharge depth, the resistance *R*_b_ of FNC gradually decreases after fitting (Figure 5C), attributed to the enhanced conductivity resulting from carbon formation during the reaction. As the discharge progresses, the *R*_ct_ of FNC remains relatively stable with minimal variation (Figure 5D). However, compared to the initial stage of discharge, the *R*_ct_ decreases as the reaction proceeds. This trend aligns with previous studies on CF_*x*_ material (Chen P. et al., 2023). At 100% DOD, *R*_ct_ does not rise sharply, which also confirms that CF_*x*_ material can continue to react at 1.5 V.

To further validate the aforementioned analysis of the discharge mechanism of FNC, the distribution of relaxation time (DRT) was employed to analyze the EIS impedance across various DOD and timescales (Figures 5E–H, Initiating the DRT diagnosis process involves the utilization of EIS measurement across a wide frequency spectrum) (Rashid and Gupta, 2015). Initially, it is observed that the relaxation time constant τ exhibits minimal impedance response within the interval of (−5 ≤ Logτ ≤ −1), suggesting the absence of solid electrolyte interphase (SEI) formation throughout the discharge process of FNC, thereby excluding consideration of *R*_sei_. The relaxation time constant τ falls within the interval of (0 ≤ Logτ ≤2), indicating the charge transfer process. It manifests a pronounced impedance signal during the initial discharge stage, gradually diminishing as the discharge deepens, aligning with the aforementioned analysis findings. Overall, owing to the ultra-low conductivity of CF_*x*_ material, the charge transfer impedance is predominantly influenced by conductivity.

Subsequently, we fabricated new SPBs employing the FNC as cathode material. Equation 3, proposed by Liu et al., represents the discharge mechanism of SPBs, which is similar to that of Li/CF_*x*_ primary batteries (Liu et al., 2014):

$$CF_{x}+x{Na}^{+}+e^{‐}\to C+xNaF$$ (3)

**FIGURE 6:**
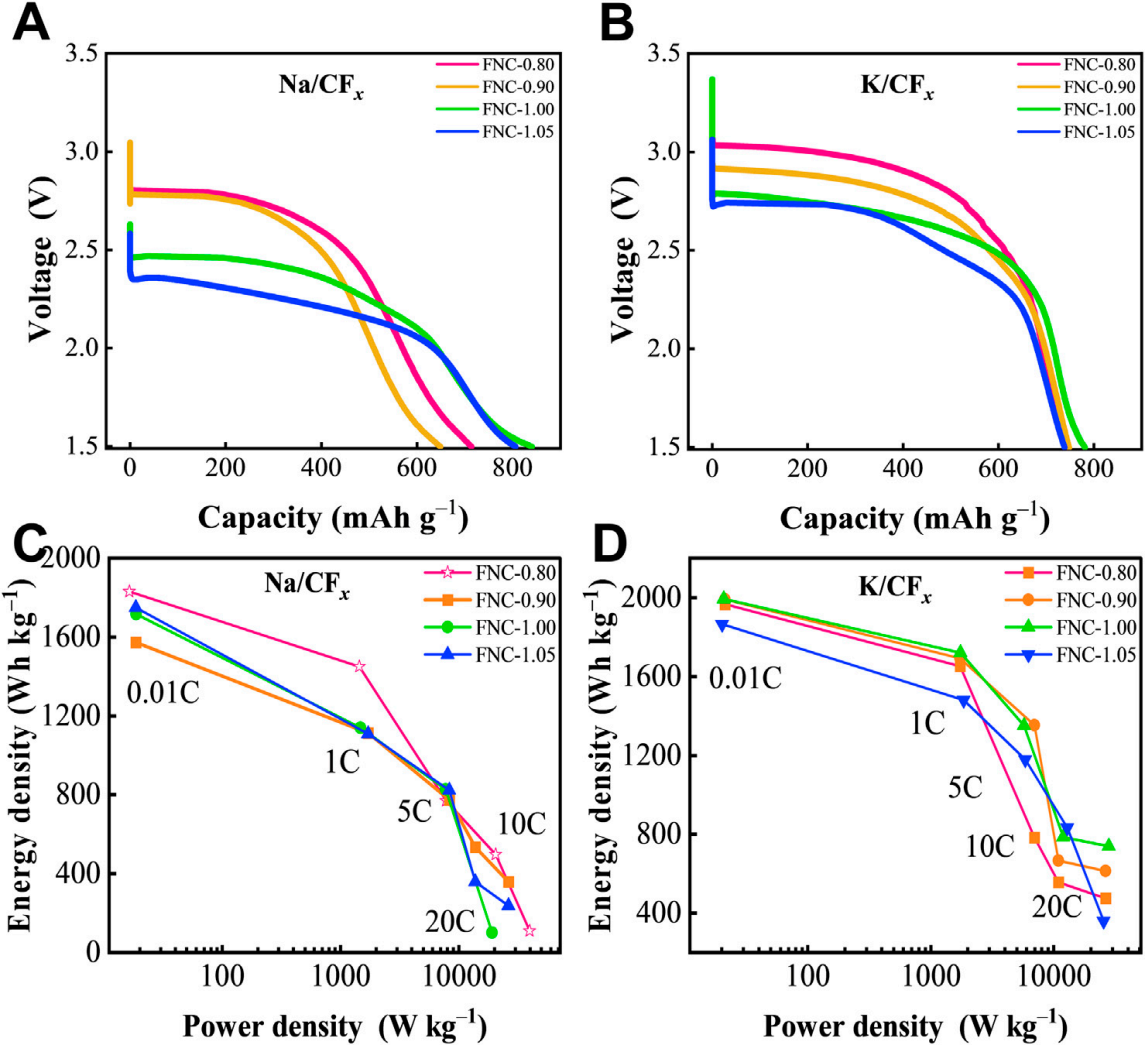
**(A, B)** Galvanostatic discharge curves of various FNC at 0.01C rate in SPBs and PPBs. **(C, D)** Diagram of energy density of the prepared FNC cathodes at different discharge rates in SPBs and PPBs. Note: Multiple batteries were simultaneously tested, and all results showed good repeatability.

Notably, the discharge voltage of FNC exhibits a notable reduction of approximately 250 mV in SPBs compared to LPBs. This discrepancy is attributed to the difference in the standard electrode potentials between lithium (Li^+^/Li, −3.02 V *v*s. SHE) and sodium (Na^+^/Na, −2.71 V *vs*. SHE). Moreover, compared to LPBs, SPBs exhibit less prominent rate capability, which is likely attributed to the larger ionic radius of Na^+^ (1.02 Å) compared to Li^+^ (0.76 Å), which results in more difficult transport of Na^+^(Li et al., 2019). Although limited by the characteristics of SPBs, FNC retains a high discharge voltage and exhibits excellent electrochemical performance, as illustrated in Figures 6A, C. Equation 4, proposed by Zhong et al., represents the discharge mechanism of PPBs, which is similar to that of the LPBs and SPBs batteries (Yue et al., 2021):

$$CF_{x}+xK^{+}+e^{-}\to C+xKF$$ (4)

As a cathode material, FNC exhibits excellent electrochemical properties in PPBs (relevant data are shown in Figures 6B, D; Supplementary Table S4). This performance can be attributed to the high standard electrode potential of potassium (K^+^/K, −2.93 V *vs*. SHE). In PPBs, FNC demonstrates high discharge voltage and excellent rate capability, with maximum capacity and energy density levels approaching those of LPBs. At a rate of 20C, it maintains sufficient stability for discharge, achieving energy density and power density of 738 Wh kg^–1^ and 22,363 W kg^–1^, respectively. The phenomenon indicates that FNC in various alkali-metal/CF_*x*_ primary batteries does not exhibit a significant voltage lag phenomenon during the initial discharge. When the discharge rate is increased to 10C or higher, stable discharge is achieved with a discharge voltage exceeding 1.5 V. This can be attributed to the favorable conductivity of FNC, which mitigates the influence of ohmic polarization. Overall, the high discharge voltage and small ohmic polarization associated with FNC enable an outstanding cathode material for alkali-metal/CF_*x*_ primary batteries. In comparison with other reported fluorinated nanocarbon materials, FNC represents the first instance of utilization in distinct alkali-metal primary batteries, demonstrating stable discharge even at high discharge rates.

The evolution of the electrode’s structural characteristics is likewise crucial. A comparative analysis of SEM imagery prior to and post discharge of the FNC-1.0 cathode before and after discharge at 0.01C in LPBs (Supplementary Figures S6A, B) reveals a large number of small particles uniformly covering the converted FNC surface. In the previously conducted studies, similar morphological characteristics were observed on the CF_*x*_ cathode after the discharge was completed, which is attributed to the contribution of small LiF crystals (Peng et al., 2019; Hu et al., 2023). In SPBs and PPBs, dense NaF and KF are also considered to generate in the FNC surface after discharge, and the morphology of the electrodes did not change much before and after discharge. Overall, the morphology of FNC is well maintained during discharge, in alignment with previous report (Cai et al., 2023).

## 4 Conclusion

In summary, we have proposed a chemical pathway for synthesizing FNC that possesses exceptional electrochemical properties, using high surface area aggerated nanocarbon materials as precursor. The FNC synthesized via fluoridation maintains the original morphology of ANC with large surface area and short Li^+^ pathway and exhibits high electric conductivity, effectively mitigating the voltage lag caused by ohmic polarization during the initial stage. With a substantial content of semi-ionic C–F bonds, FNC demonstrates exceptional rate capability. As a result, FNC-1.0 with a high F/C ratio (*x* = 1) maintains a high discharge voltage of 3.13 V, rendering it application in various scenarios. Moreover, the FNC-1.0 exhibits a specific capacity of 578 mA h g^–1^ and a capacity retention rate of 75.6% at a magnification of 20C. Furthermore, it boasts an energy density of 1,250 Wh kg^–1^ and a power density of 33,694 W kg^–1^. The FNC could also be e*x*tended to SPBs and PPBs, further demonstrating its suitability for alkali-metal primary batteries. The unique designed FNC exhibiting high discharge voltage and outstanding rate capability, possesses significant potential for supplanting commercial fluorinated graphite in the prospective CF_*x*_ market.

## Data Availability

The original contributions presented in the study are included in the article/Supplementary Material, further inquiries can be directed to the corresponding authors.

## References

[B1] Ahmad Y. Dubois M. Guerin K. Hamwi A. Flahaut E. (2017). High energy density of primary lithium batteries working with sub-fluorinated few walled carbon nanotubes cathode. J. Alloys Compd. 726, 852–859. 10.1016/j.jallcom.2017.08.001

[B2] Ahmad Y. Dubois M. Guérin K. Hamwi A. Zhang W. (2015). Pushing the theoretical limit of Li–CFx batteries using fluorinated nanostructured carbon nanodiscs. Carbon 94, 1061–1070. 10.1016/j.carbon.2015.07.073

[B3] Cai S. Meng W. Tian H. Luo T. Wang L. Li M. (2023). Artificial porous heterogeneous interface for all-solid-state sodium ion battery. J. Colloid Interface Sci. 632, 179–185. 10.1016/j.jcis.2022.11.037 36413944

[B4] Chen N. G. Zhang G. J. Chen H. X. Yue H. J. (2023). Conductive carbon-wrapped fluorinated hard carbon composite as high-performance cathode for primary lithium batteries. Coatings 13, 812. 10.3390/coatings13050812

[B5] Chen P. Liu W. Wang H. Jiang Y. Niu X. Wang L. (2023). Semi-Ionic C-F bond enabling fluorinated carbons rechargeable as Li-ion batteries cathodes. J. Colloid Interface Sci. 649, 255–263. 10.1016/j.jcis.2023.06.108 37348345

[B6] Deiss E. (2005). Spurious chemical diffusion coefficients of Li+ in electrode materials evaluated with GITT. Electrochimica Acta 50, 2927–2932. 10.1016/j.electacta.2004.11.042

[B7] Dubois M. Guérin K. Zhang W. Ahmad Y. Hamwi A. Fawal Z. (2012). Tuning the discharge potential of fluorinated carbon used as electrode in primary lithium battery. Electrochimica Acta 59, 485–491. 10.1016/j.electacta.2011.11.015

[B8] Guérin K. Dubois M. Houdayer A. Hamwi A. (2012). Applicative performances of fluorinated carbons through fluorination routes: a review. J. Fluor. Chem. 134, 11–17. 10.1016/j.jfluchem.2011.06.013

[B9] Guérin K. Pinheiro J. Dubois M. Fawal Z. Masin F. Yazami R. (2004). Synthesis and characterization of highly fluorinated graphite containing sp2 and sp3 carbon. Chem. Mater. 16, 1786–1792. 10.1021/cm034974c

[B10] Hou J. Yang X. Fu X. Zou D. Ma J. Peng Y. (2022). Highly oriented fluorinated carbon nanotube arrays for high specific capacity lithium primary battery. J. Alloys Compd. 923, 166452. 10.1016/j.jallcom.2022.166452

[B11] Hu Y. H. Kong L. C. Li W. Y. Sun L. D. Peng C. Qin M. (2023). Fluorinated microporous carbon spheres for Li/CF_x_ batteries with high volumetric energy density. Compos. Commun. 40, 101607. 10.1016/j.coco.2023.101607

[B12] Jayasinghe R. Thapa A. K. Dharmasena R. R. Nguyen T. Q. Pradhan B. K. Paudel H. S. (2014). Optimization of Multi-Walled Carbon Nanotube based CF electrodes for improved primary and secondary battery performances. J. Power Sources 253, 404–411. 10.1016/j.jpowsour.2013.12.076

[B13] Jiang H. R. Shyy W. Wu M. C. Wei L. Zhao T. S. (2017). Highly active, bi-functional and metal-free B 4 C-nanoparticle-modified graphite felt electrodes for vanadium redox flow batteries. J. Power Sources 365, 34–42. 10.1016/j.jpowsour.2017.08.075

[B14] Jiang S. B. Huang P. Lu J. C. Liu Z. C. (2021). The electrochemical performance of fluorinated ketjenblack as a cathode for lithium/fluorinated carbon batteries. RSC Adv. 11, 25461–25470. 10.1039/d1ra03873g 35478916 PMC9036963

[B15] Jones J. P. Smart M. C. Krause F. C. West W. C. Brandon E. J. (2022). Batteries for robotic spacecraft. Joule 6, 923–928. 10.1016/j.joule.2022.04.004

[B16] Lee Y. S. Cho T. H. Lee B. K. Rho J. S. An K. H. Lee Y. H. (2003). Surface properties of fluorinated single-walled carbon nanotubes. J. Fluor. Chem. 120, 99–104. 10.1016/s0022-1139(02)00316-0

[B17] Leifer N. Johnson V. Ben-Ari R. Gan H. Lehnes J. Guo R. (2009). Solid-state NMR studies of chemically lithiated CF_x_ . J. Electrochem. Soc. 157, A148. 10.1149/1.3267042 PMC2911803 20676233

[B18] Li X. Zhang H. Liu C. Qiao J. Zhou X. (2021). A MOF-derived multifunctional nano-porous fluorinated carbon for high performance lithium/fluorinated carbon primary batteries. Microporous Mesoporous Mater. 310, 110650. 10.1016/j.micromeso.2020.110650

[B19] Li Y. Wu X. Liu C. Wang S. Zhou P. Zhou T. (2019). Fluorinated multi-walled carbon nanotubes as cathode materials of lithium and sodium primary batteries: effect of graphitization of carbon nanotubes. J. Mater. Chem. A 7, 7128–7137. 10.1039/c8ta12074a

[B20] Liu W. Li H. Xie J.-Y. Fu Z.-W. (2014). Rechargeable room-temperature CF_x_-sodium battery. ACS Appl. Mater. and Interfaces 6, 2209–2212. 10.1021/am4051348 24494989

[B21] Liu Y. Zhang H. Wu B. Ma J. Zhou G. Mahmood N. (2023). Pushing capacities and energy densities beyond theoretical limits of lithium primary batteries using active CF_x_ nanocapsules with *x* > 1. Inorg. Chem. Front. 10, 127–136. 10.1039/d2qi02027k

[B22] Lu Y. Ru S. Li H. Wang G. Xu S. (2023). Laser-structured microarray electrodes for durable stretchable lithium-ion battery. J. Colloid Interface Sci. 631, 1–7. 10.1016/j.jcis.2022.11.024 36379111

[B23] Nair R. R. Ren W. Jalil R. Riaz I. Kravets V. G. Britnell L. (2010). Fluorographene: a two‐dimensional counterpart of teflon. Small 6, 2877–2884. 10.1002/smll.201001555 21053339

[B24] Peng C. Li Y. Yao F. Fu H. Zhou R. Feng Y. (2019). Ultrahigh-energy-density fluorinated calcinated macadamia nut shell cathodes for lithium/fluorinated carbon batteries. Carbon 153, 783–791. 10.1016/j.carbon.2019.07.065

[B25] Peng C. Zhang S. Kong L. Xu H. Li Y. Feng W. (2023). Fluorinated carbon nanohorns as cathode materials for ultra‐high power Li/CF_x_ batteries. Small Methods 8, 2301090. 10.1002/smtd.202301090 38009765

[B26] Qian M. Tang Y. Liu L. Gao Y. Li X. (2022). Well-dispersed Li2CoTi3O8 nanoparticles as a multifunctional material for lithium-ion batteries and lithium-sulfur batteries. J. Alloys Compd. 896, 162926. 10.1016/j.jallcom.2021.162926

[B27] Rajagopal R. R. Rajarao R. Sahajwalla V. (2017). Synthesis of glass fiber-nano silicon carbide composite by using waste printed circuit boards and compact discs as resources. Compos. Commun. 5, 19–22. 10.1016/j.coco.2017.05.002

[B28] Ramachandran T. Hamed F. Raji R. K. Majhi S. M. Barik D. Kumar Y. A. (2023). Enhancing asymmetric supercapacitor performance with NiCo_2_O_4_–NiO hybrid electrode fabrication. J. Phys. Chem. Solids 180, 111467. 10.1016/j.jpcs.2023.111467

[B29] Ramachandran T. Mourad A. H. I. Raji R. K. Krishnapriya R. Cherupurakal N. Subhan A. (2022). KOH mediated hydrothermally synthesized hexagonal‐CoMn_2_O_4_ for energy storage supercapacitor applications. Int. J. Energy Res. 46, 16823–16838. 10.1002/er.8350

[B30] Rashid M. Gupta A. (2015). Effect of relaxation periods over cycling performance of a Li-ion battery. J. Electrochem. Soc. 162, A3145–A3153. 10.1149/2.0201502jes

[B31] Robinson J. T. Burgess J. S. Junkermeier C. E. Badescu S. C. Reinecke T. L. Perkins F. K. (2010). Properties of fluorinated graphene films. Nano Lett. 10, 3001–3005. 10.1021/nl101437p 20698613

[B32] Sato Y. Itoh K. Hagiwara R. Fukunaga T. Ito Y. (2004). On the so-called “semi-ionic” C–F bond character in fluorine–GIC. Carbon 42, 3243–3249. 10.1016/j.carbon.2004.08.012

[B33] Sharma N. Dubois M. Guérin K. Pischedda V. Radescu S. (2021). Fluorinated (Nano)Carbons: CF_x_ electrodes and CF_x_‐based batteries. Energy Technol. 9, 2000605. 10.1002/ente.202000605

[B34] Sun C. B. Feng Y. Y. Li Y. Qin C. Q. Zhang Q. Q. Feng W. (2014). Solvothermally exfoliated fluorographene for high-performance lithium primary batteries. Nanoscale 6, 2634–2641. 10.1039/c3nr04609e 24336908

[B35] Wang D. Jia X. Tian R. Yang J. Su Y. Song H. (2024). Tuning fluorine content of fluorinated graphene by an ionothermal synthesis method for achieving excellent tribological behaviors. Carbon 218, 118649. 10.1016/j.carbon.2023.118649

[B36] Watanabe N. (1980). Two types of graphite fluorides, (CF)n and (C2F)n, and discharge characteristics and mechanisms of electrodes of (CF)n and (C2F)n in lithium batteries. Solid State Ionics 1, 87–110. 10.1016/0167-2738(80)90025-9

[B37] Xu Z. Buehler M. J. (2009). Strain controlled thermomutability of single-walled carbon nanotubes. Nanotechnology 20, 185701. 10.1088/0957-4484/20/18/185701 19420624

[B38] Yang X. X. Zhang G. J. Bai B. S. Li Y. Li Y. X. Yang Y. (2021). Fluorinated graphite nanosheets for ultrahigh-capacity lithium primary batteries. Rare Met. 40, 1708–1718. 10.1007/s12598-020-01692-y

[B39] Yazami R. Hamwi A. Guérin K. Ozawa Y. Dubois M. Giraudet J. (2007). Fluorinated carbon nanofibres for high energy and high power densities primary lithium batteries. Electrochem. Commun. 9, 1850–1855. 10.1016/j.elecom.2007.04.013

[B40] Yue H. Chen H. Zhao C. Zheng Z. Zhou K. Zhang Q. (2021). Reversible potassium storage in ultrafine CF: a superior cathode material for potassium batteries and its mechanism. J. Energy Chem. 53, 347–353. 10.1016/j.jechem.2020.05.024

[B41] Zhang Q. D’Astorg S. Xiao P. Zhang X. Lu L. (2010). Carbon-coated fluorinated graphite for high energy and high power densities primary lithium batteries. J. Power Sources 195, 2914–2917. 10.1016/j.jpowsour.2009.10.096

[B42] Zhang S. S. Foster D. Read J. (2009a). Carbothermal treatment for the improved discharge performance of primary Li/CFx battery. J. Power Sources 191, 648–652. 10.1016/j.jpowsour.2009.02.007

[B43] Zhang S. S. Foster D. Wolfenstine J. Read J. (2009b). Electrochemical characteristic and discharge mechanism of a primary Li/CFx cell. J. Power Sources 187, 233–237. 10.1016/j.jpowsour.2008.10.076

[B44] Zhong G. Chen H. Huang X. Yue H. Lu C. (2018). High-power-density, high-energy-density fluorinated graphene for primary lithium batteries. Front. Chem. 6, 50. 10.3389/fchem.2018.00050 29594098 PMC5854643

[B45] Zhu C. Shen X. Gao Z. Li Y. Wu X. Zhao J. (2022). Enhancing electrochemical performance of fluorinated graphite by polydopamine−derived nitrogen−doped carbon coating. Electrochimica Acta 425, 140718. 10.1016/j.electacta.2022.140718

[B46] Zhu D. Yuan J. Wang T. Dai Y. Peng Y. Li W. (2022). A novel one-step method to prepare N, S Co-doped sub-fluorinated carbon electrode materials for ultrahigh-rate lithium-fluorinated carbon battery. J. Power Sources 551, 232188. 10.1016/j.jpowsour.2022.232188

[B47] Zou Y. Yan K. Bao L. Xia Q. Chen H. Yue H. (2024). Fluorinated hollow porous carbon spheres as high-performance cathode material for primary battery. Batteries 10, 310. 10.3390/batteries10090310

