# Supplementary material for: Fluorinated aggregated nanocarbon with high discharge voltage as cathode materials for alkali-metal primary batteries
Source: Front Chem. 2024 Oct 2;12:1484668. doi: 10.3389/fchem.2024.1484668 (PMC11479893; doi:10.3389/fchem.2024.1484668)
Supplement: Supplementary file 1 [file DataSheet1.docx]

Supplementary Material

**Fluorinated aggregated nanocarbon with high discharge voltage as cathode materials for alkali-metal primary batteries**

Huixin Chen^1,2,4,#^, Ke Yan^2,3,4,#^, Yan Zou^2,3,4^, Qi Xia^2,4^, Xiaoyu Kang^5^, Hongjun Yue^2,4,*^, Ding Chen^1,*^

^1^ State Key Laboratory of Advanced Design and Manufacturing for Vehicle Body, College of Mechanical and Vehicle Engineering, Hunan University, Changsha 410082, Hunan, China

^2^ State Key Laboratory of Structural Chemistry, Fujian Institute of Research on the Structure of Matter, Chinese Academy of Sciences, Fuzhou 350002, Fujian, China

^3^ College of Chemistry, Fuzhou University, Fuzhou 350108, Fujian, China

^4^ Xiamen Key Laboratory of Rare Earth Photoelectric Functional Materials, Xiamen Institute of Rare Earth Materials, Haixi institutes, Chinese Academy of Sciences, Xiamen 361021, Fujian, China

^5^ Changsha Ecological Environmental Monitoring Centre of Hunan Province, Changsha 410001, Hunan, China

^#^ These authors contributed equally to this work.

^*^ Corresponding authors.

E-mail address:

hjyue@fjirsm.ac.cn; [chending@hnu.edu.cn](mailto:chending@hnu.edu.cn);

**Supplementary Figures**

**
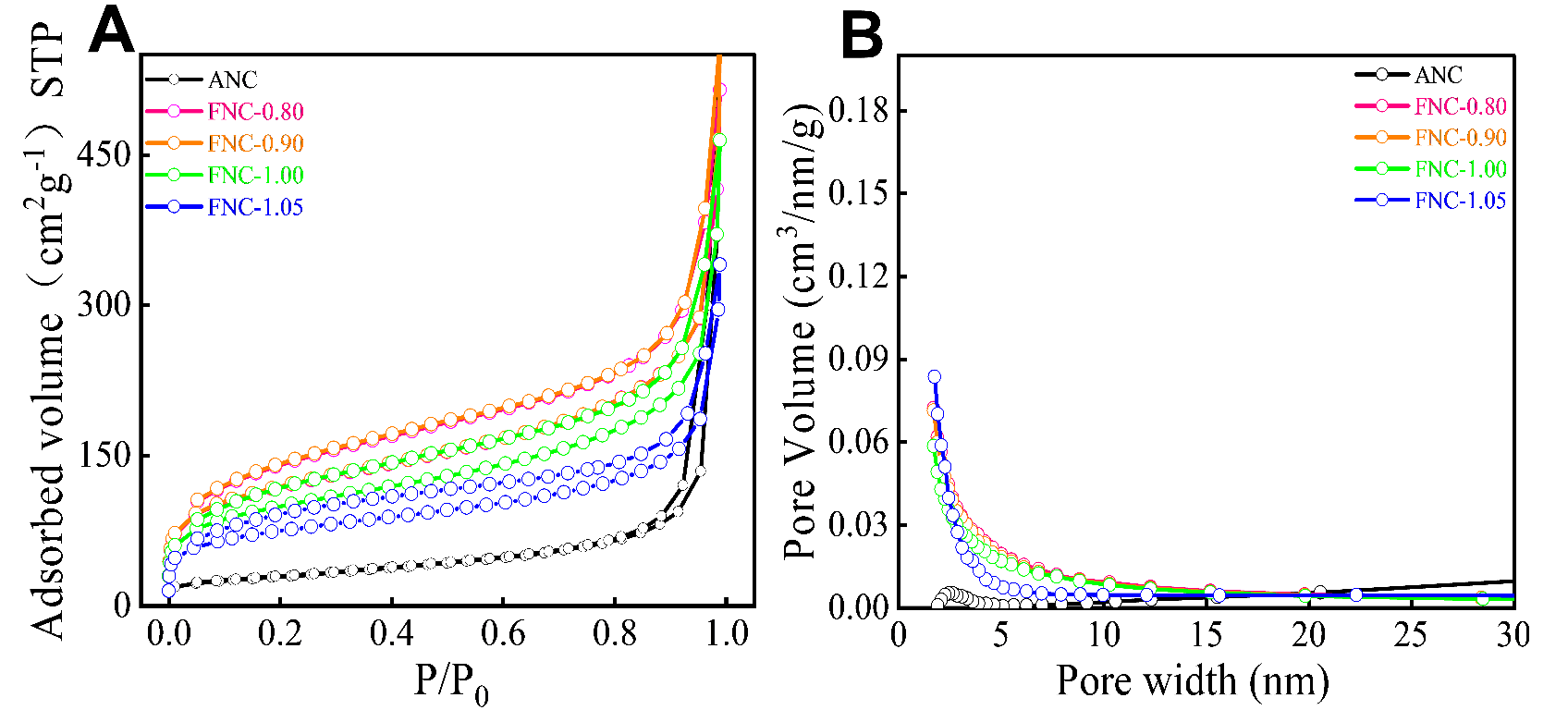
**

**Supplementary Figure S1.** (A) adsorption-desorption isotherms of NC and FNC, (B) N_2_ Pore size distribution of NC and FNC.


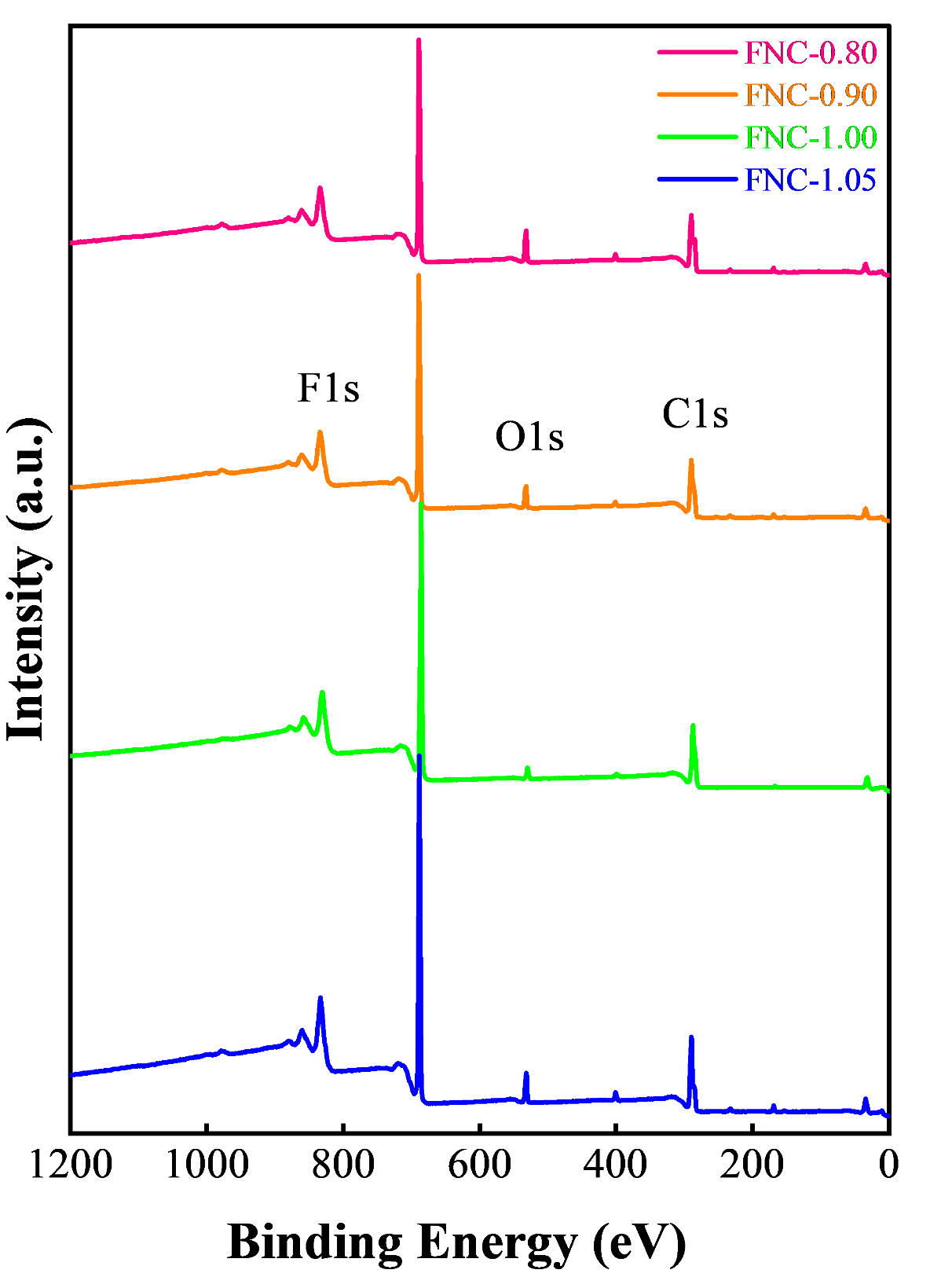


**Supplementary Figure S2.** The XPS survey spectra of FNC


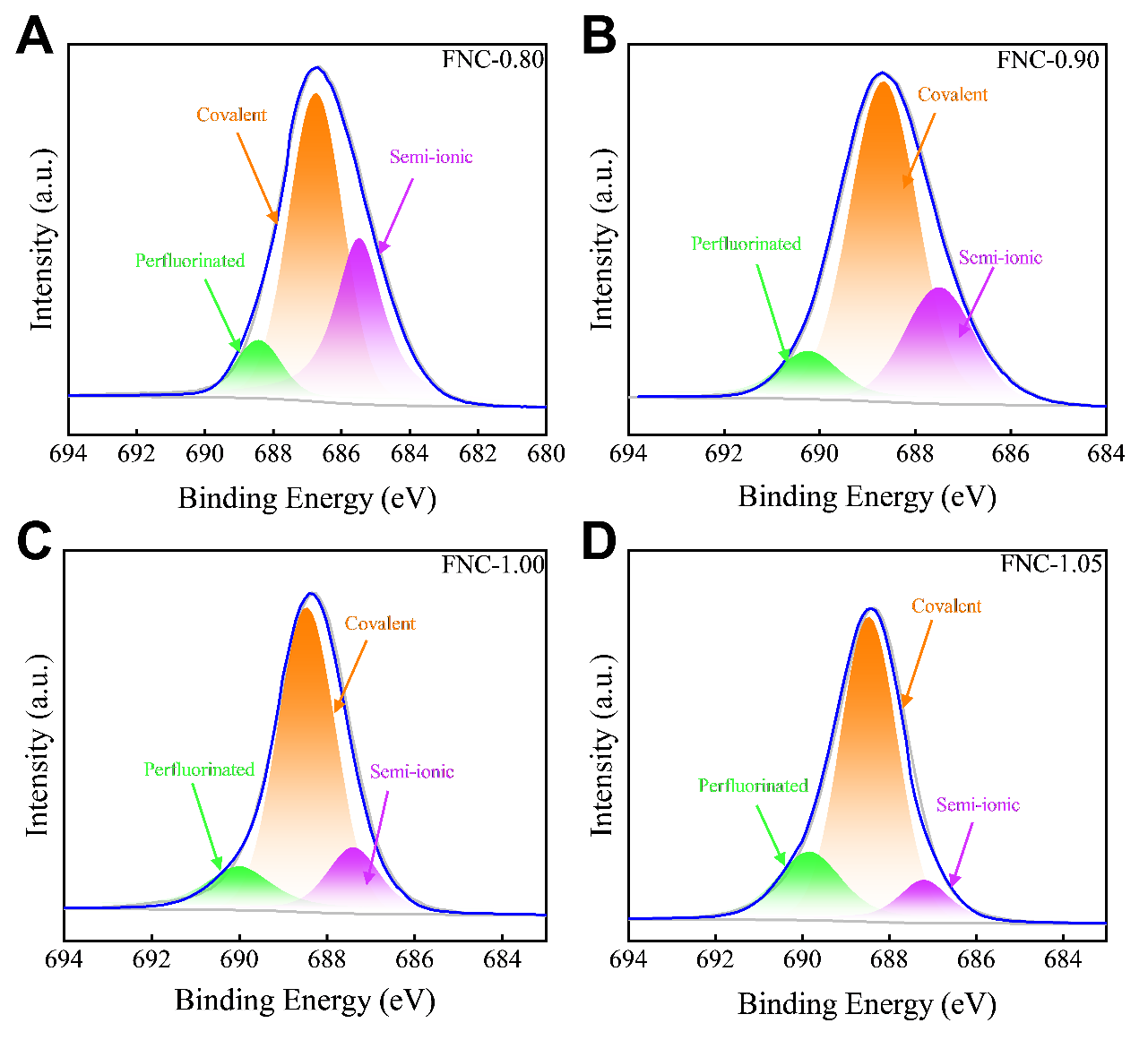


**Supplementary Figure S3.** Comparative XPS F 1s spectra of (**A**) FNC-0.80, (B) FNC-0.90, (C) FNC-1.00, (D) FNC-1.05.


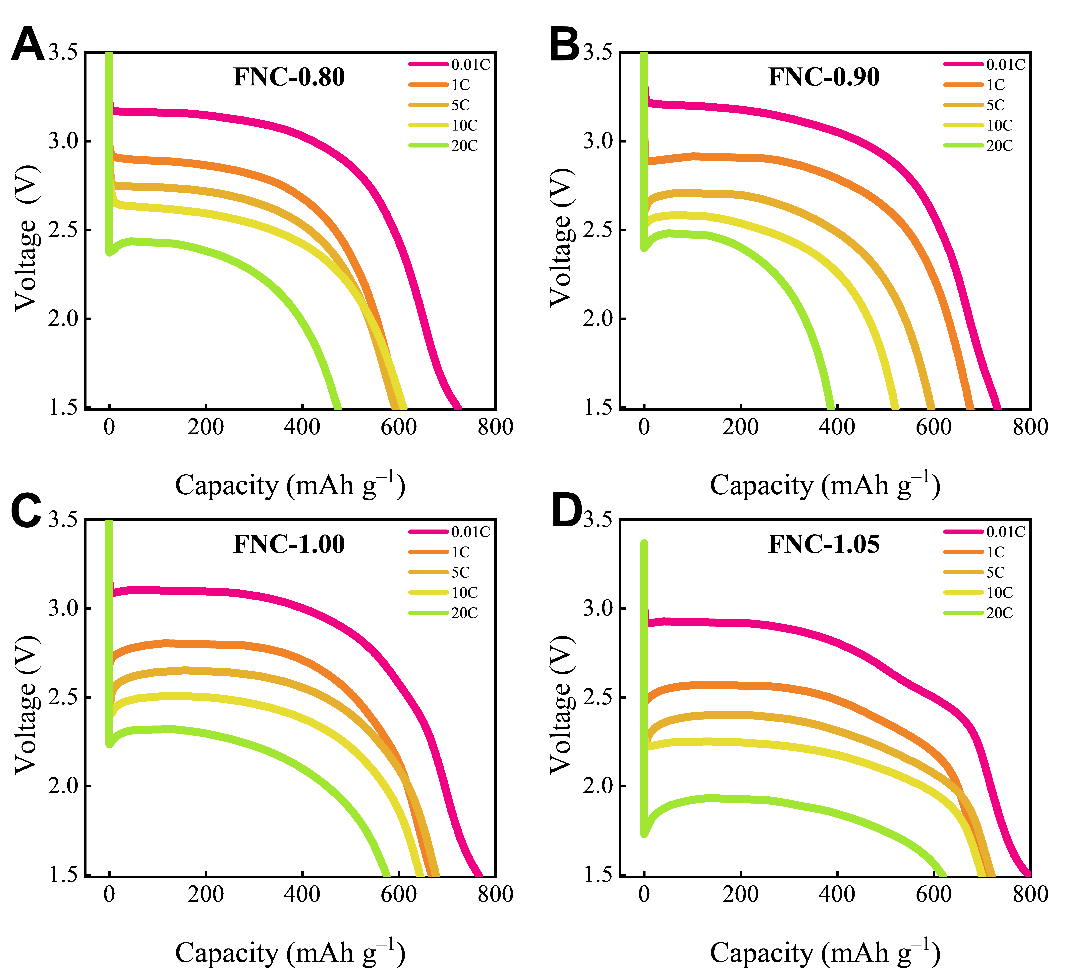


**Supplementary Figure S4** Galvanostatic discharge curves of (A) FNC-0.80, (B) FNC-0.90, (C) FNC-1.00, (D) FNC-1.05.


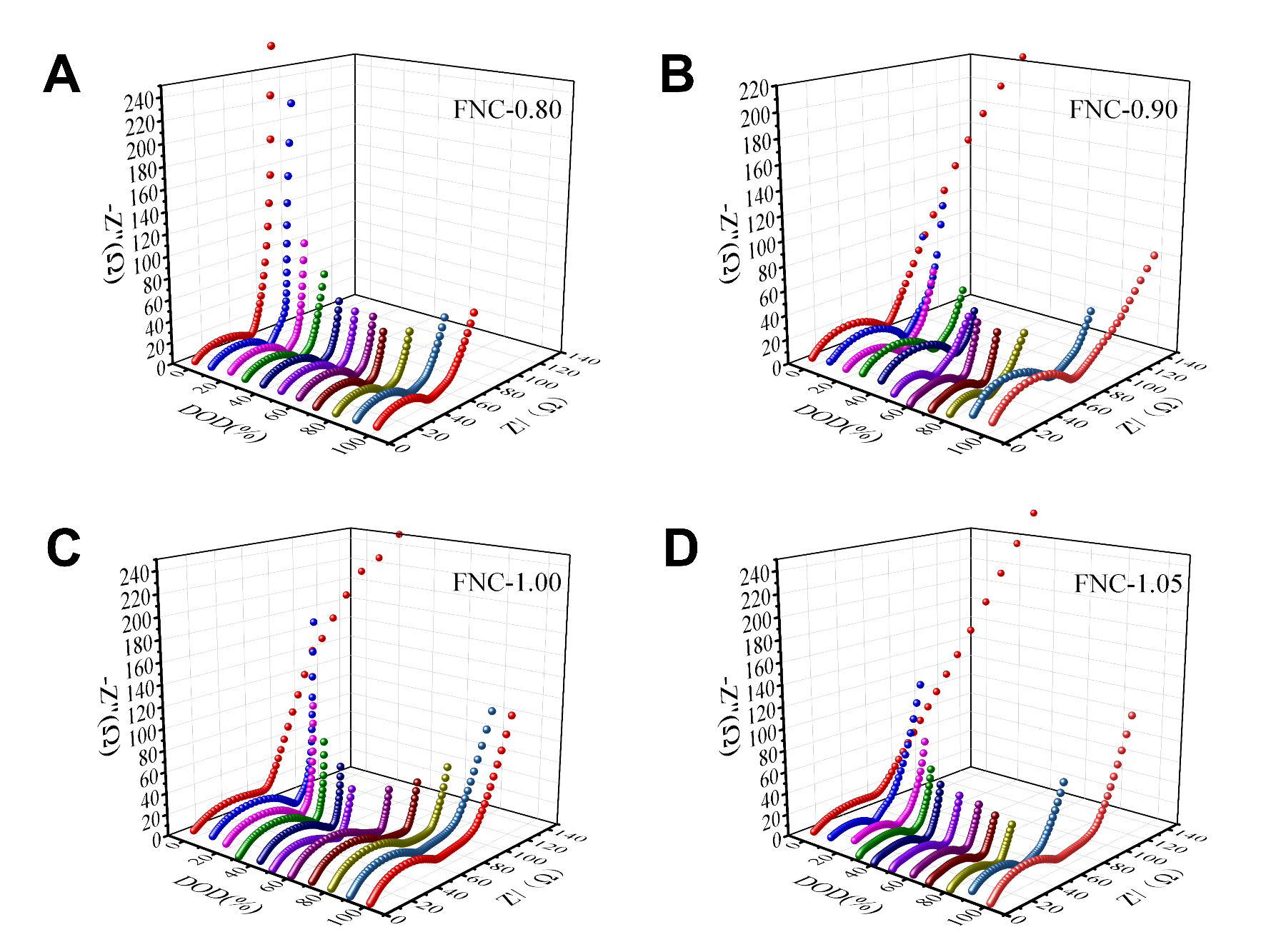


**Supplementary Figure S5****.** EIS spectra of (A) FNC-0.80 (B) FNC-0.90 (C) FNC-1.00 (D) FNC-1.05 at different.


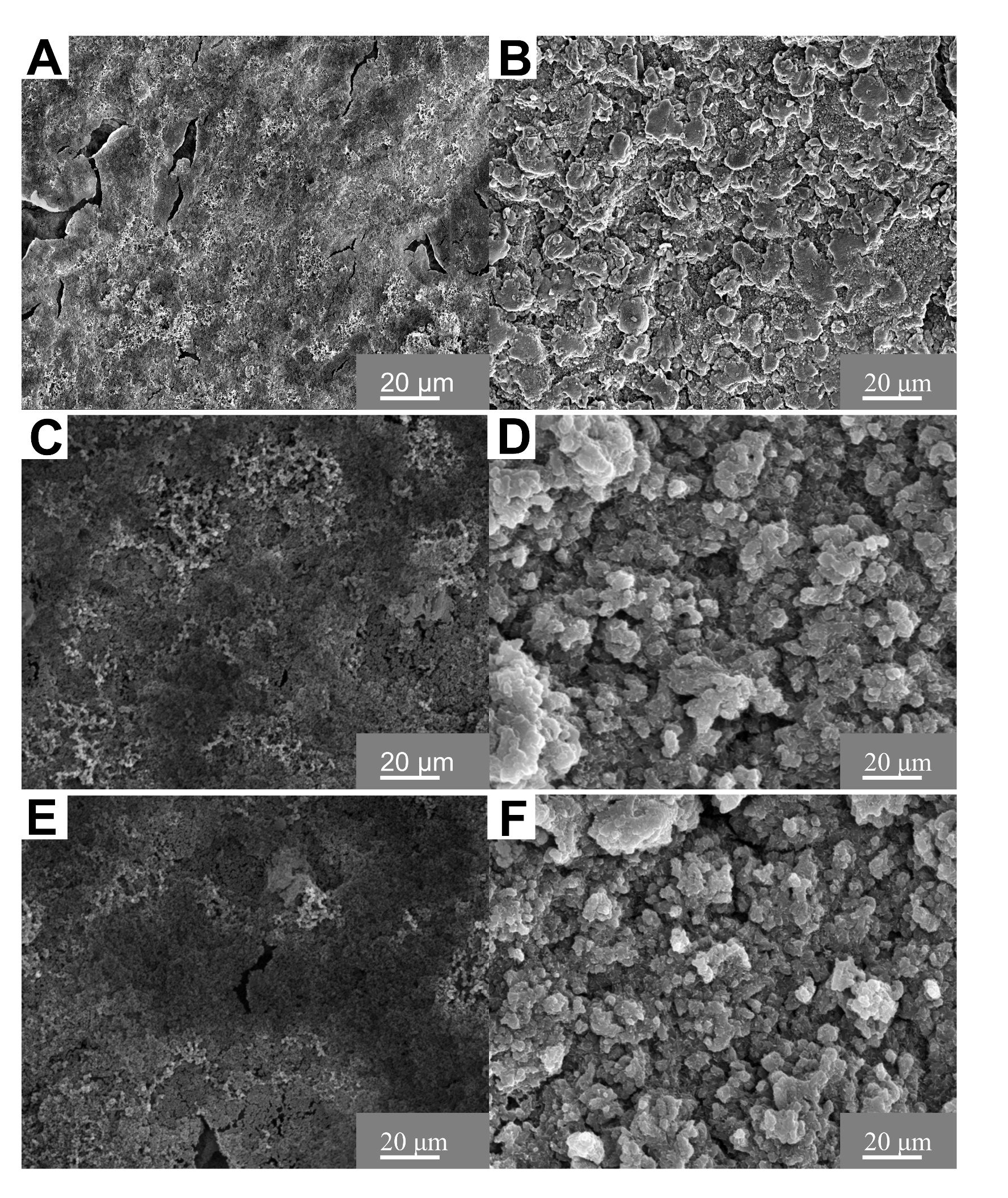


**Supplementary Figure S6** The SEM images show the morphology of FNC as a Li/CF*x* primary battery anode material before (A) and after (B) discharge. The SEM images show the morphology of FNC as a Na/CF*x* primary battery anode material before (C) and after (D) discharge. The SEM images show the morphology of FNC as a K/CF*_x_* primary battery anode material before (E) and after (F) discharge.

**Supplementary Tables**

**Supplementary Table S1** Calculated parameters from the nitrogen adsorption of NC and FNC.

| **Sample** | **BET**  **Surface**  **Area（m^2^g^-1^）** | **Total**  **Pore volume**  **(m^3^g^-1^)** | **Micropore**  **Volume**  **(m^3^g^-1^)** | **Pore width**  **（nm）** |
| --- | --- | --- | --- | --- |
| **NC** | 104.062 | 0.857 | 0.003 | 0.6010 |
| **FNC-0.80** | 424.388 | 0.796 | 0.042 | 0.642 |
| **FNC-0.90** | 426.369 | 0.871 | 0.044 | 0.636 |
| **FNC-1.0** | 355.871 | 0.718 | 0.035 | 0.637 |
| **FNC-1.05** | 270.364 | 0.526 | 0.032 | 0.603 |

**Supplementary Table S2** Composition contents and calculated F/C values of NC and FNC.

| **Sample** | **C** | **F** | **O** | **F/C** |
| --- | --- | --- | --- | --- |
|  | **(at%)** | **(at%)** | **(at%)** |  |
| **FNC-0.80** | 53.21 | 44.1 | 2.69 | 0.828 |
| **FNC-0.90** | 51.05 | 45.95 | 3.0 | 0.90 |
| **FNC-1.00** | 49.55 | 47.85 | 2.6 | 0.966 |
| **FNC-1.05** | 48.34 | 49.35 | 2.31 | 1.02 |

**Supplementary Table S3** C1s peaks assignments and proportions, and F1s peaks assignments and proportions obtained from the XPS spectra of FNC.

| **Sample** |  |  | **C1s assignment (eV)** |  |  |  |  | **F1s assignment (eV)** | |
| --- | --- | --- | --- | --- | --- | --- | --- | --- | --- |
|  | **C=C** | **C-C** | **Semi-ionic**  **C–F** | **Covalent**  **C–F** | **CF_2_** | **CF_3_** | **Semi-ionic (C_x_F)_n_** | **Covalent (CF)_n_** | **Perfluorinated-CF_2_/CF_3_** |
|  | **(%)** | **(%)** | **(%)** | **(%)** | **(%)** | **(%)** | **(%)** | **(%)** | **(%)** |
| **FNC-0.80** | 284.8 | 286.57 | 288.45 | 289.68 | 291.1 | 292.15 | 687.92 | 689.53 | 691.39 |
|  | 38.37 | 0.01 | 26.75 | 21.34 | 10.36 | 3.17 | 25.31 | 62.83 | 11.86 |
| **FNC-0.90** | 284.78 | 287.11 | 289.53 | 290.14 | 291.89 | 292.98 | 687.31 | 688.96 | 690.03 |
|  | 13.31 | 17.58 | 12.20 | 42.98 | 8.31 | 5.62 | 24.39 | 61.34 | 14.27 |
| **FNC-1.00** | 284.8 | 287.28 | 289.21 | 290.32 | 291.95 | 292.87 | 687.40 | 688.68 | 689.99 |
|  | 18.31 | 14.39 | 9.31 | 41.32 | 10.31 | 6.36 | 21.39 | 58.46 | 20.15 |
| **FNC-1.05** | 284.75 | 286.49 | 288.76 | 289.44 | 291.05 | 291.99 | 687.00 | 688.43 | 689.37 |
|  | 11.21 | 9.33 | 8.54 | 46.02 | 14.59 | 10.31 | 18.78 | 51.56 | 29.66 |

**Supplementary Table S4** Comparison of the discharge performances of the FNC products in this study and previously reported CF*x* cathodes.

| **Reference(text)** | **Materials** | **F/C** | **Potential *E*_1/2_ (V) /**  **Discharge rate(C)** | **Maximum energy density (Wh kg^–1^) /**  **Discharge rate(C)** | **Maximum** **power**  **density (W kg^–1^) /**  **Discharge rate(C)** |
| --- | --- | --- | --- | --- | --- |
| Jiang, et al | Fluorinated ketjenblack | 0.87 | 3.03/0.01 | 2544/0.01 | 27493/20 |
| Ahmad, et al | Fluorinated carbon nanodiscs | 0.95 | 2.39/0.01 | 2338/0.01 | / |
| Li, et al | Fluorinated Multi-Walled Carbon Nanotubes | 0.81 | ~2.68/0.01 | 2006.6/0.01 | 3861/2 |
| Yang, et al | Fluorinated graphite nanosheets | 1.34 | ~2.47/0.01 | 2210/0.01 | 432/0.2 |
| Liu, et al | Nanocapsules | 1.61 | 2.75/0.01 | 2478/0.01 | / |
| Li, et al | MOF-derived multifunctional nano-porous fluorinated carbon | 0.94 | 2.70/0.01 | 2110.7/0.01 | 6540/3 |
| Peng, et al | Fluorinated Carbon Nanohorns | 1.11 | ~2.70/0.05 | 2231/0.05 | 92500/50 |
| Ahmad, et al | sub-Fluorinated few walled carbon nanotubes cathode | 0.4 | 2.73/0.01 | 2565/0.01 | / |
| Hou, et al | Fluorinated carbon nanotube arrays | 2.11 | ~2.45/0.01 | 1754/0.01 | / |
| **This work** | **FNC-0.80** | **0.828** | **3.20/0.01** | **2053/0.01** | **38823/20** |
| **This work** | **FNC-0.90** | **0.90** | **3.21/0.01** | **2113/0.01** | **34619/20** |
| **This work** | **FNC-1.00** | **0.966** | **3.13/0.01** | **2144/0.01** | **33694/20** |
| **This work** | **FNC-1.05** | **1.02** | **2.88/0.01** | **2103/0.01** | **29051/20** |

**Supplementary Table S5** The electrochemical properties of FNC as a cathode in alkali metal /CF*x* primary cells

| Sample | Primary battery type | | Discharge rate | Specific capacity  (mAh g^-1^) | Energy density  (Wh kg^-1^) |
| --- | --- | --- | --- | --- | --- |
| FNC-0.80 | | Li/CF*_x_* Primary battery | 0.01C | 722 | 2053 |
|  |  |  | 1C | 607 | 1603 |
|  |  |  | 5C | 592 | 1492 |
|  |  |  | 10C | 609 | 1467 |
|  |  |  | 20C | 475 | 1022 |
| FNC-0.80 | | Na/CF*_x_* Primary battery | 0.01C | 713 | 1749 |
|  |  |  | 1C | 495 | 1109 |
|  |  |  | 5C | 409 | 823 |
|  |  |  | 10C | 204 | 359 |
|  |  |  | 20C | 151 | 237 |
| FNC-0.80 | | K/CF*_x_* Primary battery | 0.01C | 738 | 1966 |
|  |  |  | 1C | 650 | 1652 |
|  |  |  | 5C | 336 | 782 |
|  |  |  | 10C | 253 | 557 |
|  |  |  | 20C | 220 | 474 |
| FNC-0.90 | | Li/CF*_x_* Primary battery | 0.01C | 731 | 2113 |
|  |  |  | 1C | 675 | 1817 |
|  |  |  | 5C | 531 | 1308 |
|  |  |  | 10C | 520 | 1277 |
|  |  |  | 20C | 389 | 894 |
| FNC-0.90 | | Na/CF*_x_* Primary battery | 0.01C | 648 | 1573 |
|  |  |  | 1C | 503 | 1114 |
|  |  |  | 5C | 395 | 774 |
|  |  |  | 10C | 291 | 534 |
|  |  |  | 20C | 204 | 359 |
| FNC-0.90 | | K/CF*_x_* Primary battery | 0.01C | 747 | 1990 |
|  |  |  | 1C | 711 | 1691 |
|  |  |  | 5C | 612 | 1353 |
|  |  |  | 10C | 300 | 666 |
|  |  |  | 20C | 283 | 613 |
| FNC-1.00 | | Li/CF*_x_* Primary battery | 0.01C | 765 | 2144 |
|  |  |  | 1C | 668 | 1735 |
|  |  |  | 5C | 676 | 1666 |
|  |  |  | 10C | 645 | 1502 |
|  |  |  | 20C | 578 | 1250 |
| FNC-1.00 | | Na/CF*_x_* Primary battery | 0.01C | 833 | 1831 |
|  |  |  | 1C | 723 | 1449 |
|  |  |  | 5C | 441 | 769 |
|  |  |  | 10C | 306 | 497 |
|  |  |  | 20C | 66 | 109 |
| FNC-1.00 | | K/CF*_x_* Primary battery | 0.01C | 781 | 1994 |
|  |  |  | 1C | 725 | 1720 |
|  |  |  | 5C | 612 | 1353 |
|  |  |  | 10C | 386 | 786 |
|  |  |  | 20C | 396 | 738 |
| FNC-1.05 | | Li/CF*_x_* Primary battery | 0.01C | 798 | 2103 |
|  |  |  | 1C | 703 | 1689 |
|  |  |  | 5C | 700 | 1489 |
|  |  |  | 10C | 617 | 1134 |
|  |  |  | 20C | 355 | 617 |
| FNC-1.05 | | Na/CF*_x_* Primary battery | 0.01C | 805 | 1716 |
|  |  |  | 1C | 615 | 1140 |
|  |  |  | 5C | 494 | 828 |
|  |  |  | 10C | 62 | 102 |
|  |  |  | 20C | / | / |
| FNC-1.05 | | K/CF*_x_* Primary battery | 0.01C | 731 | 1865 |
|  |  |  | 1C | 670 | 1480 |
|  |  |  | 5C | 581 | 1180 |
|  |  |  | 10C | 439 | 834 |
|  |  |  | 20C | 175 | 360 |
